# Mixed Methods Study Protocol: Language Identity, Discrimination, and Mental Health among Multilingual 1.5 Generation Asian/Asian American Immigrant Young Adults

**DOI:** 10.3390/ijerph21101311

**Published:** 2024-09-30

**Authors:** Chulwoo Park, Mark Edberg, Janet Yougi Bang, Avizia Yim Long

**Affiliations:** 1 Department of Public Health and Recreation, San José State University, San Jose, CA 95192, USA; 2 Department of Prevention and Community Health, Milken Institute School of Public Health, George Washington University, Washington, DC 20052, USA; medberg@gwu.edu; 3 Department of Child and Adolescent Development, San José State University, San Jose, CA 95192, USA; janet.bang@sjsu.edu; 4 Department of World Languages and Literatures, San José State University, San Jose, CA 95192, USA; avizia.long@sjsu.edu

**Keywords:** 1.5 generation, language proficiency, language identity, mental health, mixed methods study, health disparity

## Abstract

Language identity, an understudied factor, can influence isolation and discrimination, leading to disparities in well-being and mental health among immigrants. This study aims to investigate the role of language identity on structural racism and discrimination among 1.5 generation Asian/Asian American immigrants in a diverse U.S. state. We developed a three-step sequential approach: Stage 1—qualitative analysis (1A, focus group discussion; 1B, in-depth interviews); Stage 2—quantitative analysis (2A, language identity measurement scale; 2B, cross-sectional online survey; 2C, multivariate multiple linear regression); Stage 3—another round of qualitative analysis (3A, follow-up in-depth chronological interviews). Therefore, this study will contribute to the field by introducing a novel three-step mixed methods approach, marking a notable improvement over conventional explanatory or exploratory sequential designs.

## 1. Background

There has been increasing attention to multilingualism as an important issue for immigration and globalization. Relative to the many home countries for Asian/Asian Americans, the U.S. is a highly pluralistic society that embraces various classes, religions, languages, gender, regions, urban/rural areas, and other identities [1]. American culture has a powerful attractive force for immigrants [2]. Individuals in culturally pluralistic societies tend to have attitudes that favor connection with other individuals and groups. Although ethnic and racial groups in a pluralistic society may remain separate from different cultures and groups, they can interact to create a new common culture and a unique society, typically referred to as the ‘melting pot’ [3,4,5]. 

Asian/Asian American immigrant young adults who came to the U.S. with their families at an early age, called ‘1.5 generation’, are one of the neglected and unsupported immigrant groups that have not been extensively researched. These young adults face unique issues with respect to identity and language. Multilingual language learning can occur at any life stage [6,7,8] among immigrant populations in the U.S. Being exposed to English as an additional language (L2) after their first language (L1) is a common pattern, and it is important to consider how language is related to complex identities in multilingual individuals [9], and specifically may shape a sense of language identity, a construct we explore in the present work. For many Asian/Asian American immigrants, as soon as the 1.5 generation arrives in the U.S., they are exposed to a new dominant language and new culture, and familiarity with this new environment is essential for them to feel accepted into U.S. society.

Although language identity is not well understood, acculturation has been a common related area of study. Acculturation involves changes in identification, social skills, attitudes, and values that immigrants experience while adapting to the host culture and is also linked to mental health issues—psychological distress, depression, and anxiety [10,11,12,13,14]. While some immigrants willingly adjust to the dominant culture, others might still be attached to their culture of origin, the minority culture, and feel that adjusting to the other culture is challenging [15]. Norton (2010) highlights that language learners’ identities are shaped by their access to social and educational resources and interactions with dominant and marginalized linguistic groups [16]. This work also introduces the concept of investment, which contrasts with traditional motivation by focusing on how learners invest in language learning to enhance their social identity [16]. This theory is related to poststructuralist views, suggesting that identity is fluid and negotiated through power dynamics, with language acquisition success depending not just on ability but also aligning with sociocultural or ethnic factors [17].

In recent decades, societal advancements and theories illustrate the numerous complexities to consider for how identities are shaped for 1.5 generation Asian/Asian Americans. Social media use among recent migrants in the era of globalization, such as exchange students or foreign students, to interact with host country nationals was predicted to lead to poorer adaptation to the host country [18]. Their attempts to connect with host nationals through social media could lead to feelings of disconnect, reduced perceived support, and misunderstandings due to language barriers, ultimately hindering their well-being and psychological adaptation [19,20,21,22]. In contemporary societies, migration, transnationalism, and intersectionality have been pivotal to human settlement [23]. For example, Kimberlé Crenshaw’s reconceptualization of race through intersectionality illustrated how multiple dimensions intersect for women of color who experience violence, racism, and sexism. This work exemplified how multiple social identities intersect and influence individuals’ experiences of oppression or privilege for marginalized communities [24]. For 1.5 generation Asian/Asian Americans, the intersection of multiple dimensions can be seen through how language interacts with other facets of identity, such as race, gender, class, ethnicity, and immigration status. Additionally, transnationalism, defined by immigrants’ new social entanglement leaving behind their former life [25], could help explore how language identity is formed through cross-border interactions and multiculturalism. 

In addition, during the COVID-19 pandemic, there was a significant increase in instances of anti-Asian violence, xenophobia, and bias against Asian/Asian American immigrants in the U.S. [26,27,28,29]. Asian Americans are often treated as foreigners by others in the dominant society, regardless of their citizenship status or how long they have lived in the U.S., a phenomenon known as the perpetual foreigner stereotype [30]. This often appears in subtle forms, such as microaggressions, including compliments on an individual’s proficiency in English or inquiries about their nationality or place of origin [31,32]. A 2023 survey by the Pew Research Center stated that one-third of Asian Americans have been told to ‘go back to their home country’, a sentiment that surged alongside the rise in anti-Asian hate crimes during the COVID-19 pandemic [33]. According to the Center for the Study of Hate & Extremism, anti-Asian hate crimes in select U.S. cities reached a record high of 369 incidents in 2021; 121 of those cases (33%) were reported in California [34]. Among selected U.S. cities, San Francisco and San Jose in California showed a notably high increase of hate crimes from 2020 to 2022, or 567% and 89%, respectively [34]. Still, hate crime incidents have been significantly under-reported due to challenges with understanding systems of reporting and cultural norms that uphold feelings to ‘keep quiet’ [35,36,37]. 

The mental health of Asian/Asian American immigrant young adults is impacted by pressure to meet high parental academic expectations, the challenges of balancing two cultures, family obligations, and experiences of discrimination or isolation [38]. Asian cultural norms stigmatize the importance of seeking mental health care, which leads to mental health programs being considered unimportant [39].

### 1.1. A Gap in Knowledge

Challenges with perceived language proficiency are associated with several mental health issues—psychotic, mood, anxiety, and post-traumatic stress disorders—in migrants [40]. Language acquisition research suggests that there are numerous ways in which language skills in one language may support the learning of another language [41,42,43,44,45]. Current research on bilingual and multilingual language development [7,8,9,46,47,48] within diverse sociocultural contexts [49,50,51,52,53] has focused on the linguistic aspects of language acquisition or positioned language as an integral part of culture. Studies have focused on the relationship between language proficiency and various forms of identity, including generic [54,55], cultural [56,57], social [58], or ethnic [59] identity across different generations of immigrants. What remains unknown, however, is how 1.5 generation individuals would begin to acculturate when they are mainly exposed to an L2 and establish a sense of language identity in their new community with a new dominant language. Additionally, less attention has been given to how individuals perceive the impact of language identity in various settings where one or more languages are used in interactions, and how this is connected to their well-being and mental health. This gap has led to a lack of understanding of the connection between the language identity and mental health of the 1.5 generation. 

Language identity has been discussed in sociolinguistic literature as one’s connection to their sense of self through the socio-cultural contexts in which they use language to engage with others [16]. The methods proposed in this article are uniquely designed to capture the role of language identity and its consequences across the multiple sociocultural contexts that 1.5 generation immigrants may face. While proficiency in a language and a sense of language identity might be related to experiencing rejection, prejudice, or discrimination within different segments of cultural and ethnic communities, there are several aspects of separation and marginalization that may be uniquely tied to language identity for 1.5 generation immigrant young adults. We hypothesize that these experiences could impose significant psychological costs and stimulate social conflict in both their own communities and the dominant society. Moreover, the construct of language identity may play a distinct role in individuals’ psychological well-being and mental health, as it can illuminate how language influences self-expression, cultural belonging, and social interactions. A strong connection to one’s own sense of language identity can foster resilience and coping strategies, helping individuals overcome the challenges of ethnocultural identity confusion, acculturative stress, and racism and discrimination. Ultimately, this understanding can inform tailored mental health interventions that resonate with their unique cultural experiences and enhance their overall well-being as shaped by their language identity.

### 1.2. Methodological Aim

Using a multiple mixed methods design, we will investigate how perceived language proficiency and language identity among 1.5 generation Asian/Asian American immigrant young adults are associated with mental health disparities. The primary methodological aim of this three-step sequential mixed methods study is to integrate qualitative depth and quantitative breadth to comprehensively explore the relationships between language identity, acculturative stress, and mental health outcomes among 1.5 generation Asian/Asian American immigrants in the United States. The proposed procedure focuses on a purposive sample of our surrounding community in one state with diverse Asian/Asian American populations. We will also examine how language identity impacts various mental health outcomes through various mediators—such as ethnic identity, acculturative stress, racism, and discrimination. Additionally, experiences of anti-Asian hate crimes, which could be inextricably tied to the perceptions of their language identity, will be explored as a moderator. 

## 2. Methods and Design

### 2.1. Study Protocol

**Figure 1:**
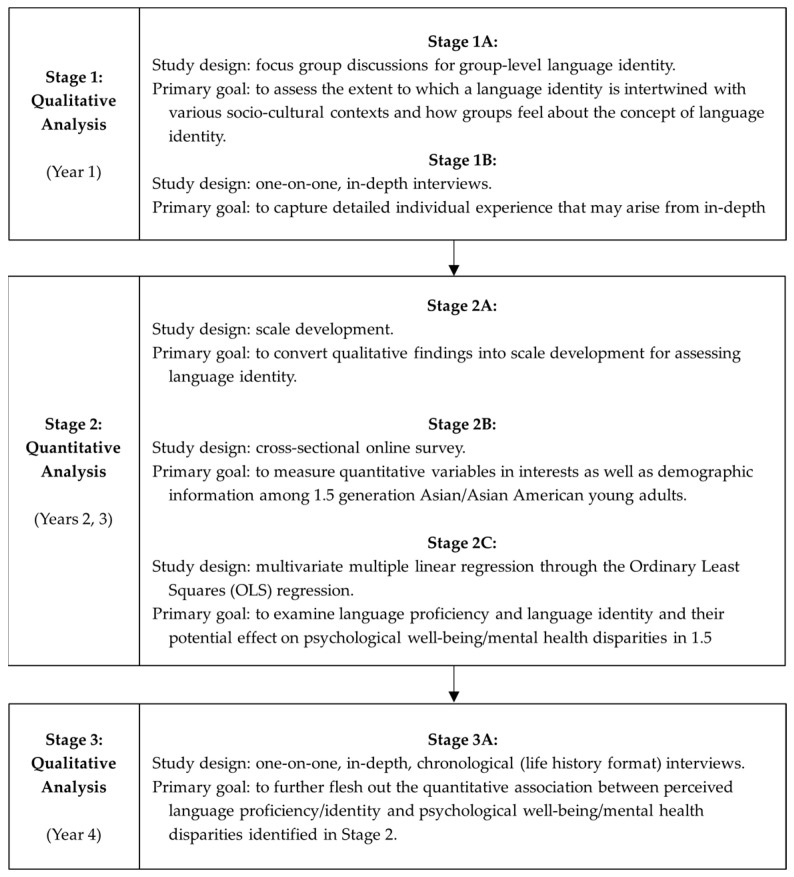
Study timeline (Stages 1–3).

The proposed mixed methods study uses a three-step sequential mixed methods approach, comprising qualitative analysis (Stage 1) and quantitative analysis (Stage 2), followed by qualitative analysis (Stage 3), over the four-year research period (Figure 1). The study protocol begins with focus group discussions (Stage 1A) to understand the background of language identity, followed by in-depth interviews (Stage 1B) to explore personal experiences with language identity. Subsequently, insights from focus group discussions and in-depth interviews will inform the development of a language identity measurement scale (Stage 2A). A cross-sectional online survey will be administered (Stage 2B), which will be analyzed using a multivariate multiple linear regression (Stage 2C). This quantitative analysis will assess how our newly developed measure of language identity, along with existing measures of perceived language proficiency, would relate to our primary outcomes through the mediator effects of ethnocultural identity confusion, acculturative stress, and racism and discrimination. We will also explore a moderator of anti-Asian hate crime victimization between language identity and our proposed mediators. The final step involves a qualitative analysis to capture additional detailed aspects from the participants, providing an in-depth understanding of how perceived language skills and language identity inform the sense of overall identity and well-being through in-depth interviews (Stage 3A). We will utilize both statistical and qualitative analysis software for this mixed methods project.

### 2.2. Target Participants

The definition of the 1.5 generation is supported by extensive literature describing individuals who describe different experiences than individuals who are considered first or second generation [60]. Individuals who are 1.5 generation are connected to cultures where ‘their heritage culture is stronger than or similar to the second-generation immigrants, but not quite to the level of first-generation immigrants either’ [61]. This group typically includes individuals who were born in their home country but migrated to new countries in elementary, intermediate, or high school [62,63]. We specified 1.5 generation Asian/Asian American immigrant young adults, our target participants in this study, as those who migrated to the U.S. with their parents (1st generation) from Asian countries when they were children or adolescents aged between 5 and 17 years, have been living in the U.S. for at least 12 months, and whose current ages are between 18 and 29 years. Our study will be conducted in California, home to 11 million immigrants (25% of the foreign-born population nationwide) with varied levels of educational attainment [64], most of whom speak at least two languages [65]. Nearly half (48.3%) of California children are living with foreign-born parents [66]. Further, more than half of recent arrivals in California (53%) between 2010 and 2019 were from Asian countries [65]. Foreign-born residents represent more than one-third of the population in 5 California counties, and 4 of them—Alameda, San Mateo, Santa Clara, and San Francisco—are located in the San Francisco Bay Area [65], where 23.3% of the population is Asian (1,664,384 out of 7,150,739) [67]. The Asian community in the Bay Area is one of the fastest-growing groups representing diverse ancestry backgrounds and cultures [68]. Thus, we will focus our study and recruitment efforts in the Bay Area, which is the main site for this research study. 

## 3. Stage 1: Qualitative Analysis

We will conduct a qualitative study to capture the meaning of language identity for Asian/Asian Americans and subsequently develop a new language identity measurement scale. In the process of developing this scale, we will delineate various socio-cultural contexts based on the country of origin that significantly contribute to an individual’s linguistic identity for 1.5 generation Asian/Asian Americans. These socio-cultural contexts encompass the home environment, educational or work settings, interactions with peers, family dynamics, and the influence of one’s occupation or professional life, as well as religious institutions. By identifying and understanding these socio-cultural contexts, we can gain valuable insights into the multifaceted nature of language identity for this population. Assessing the significance of each context allows for a nuanced exploration of how individuals prioritize and connect with different aspects of their linguistic and social environment. This exploration extends to understanding the broader sociolinguistic landscape, including the role of the home language versus English and the nuanced ways in which individuals navigate linguistic choices in different contexts. 

As part of the measurement scale development, we will investigate which specific socio-cultural context holds the highest importance for individuals, which allows for a focused understanding of the dominant factors shaping language identity. Additionally, evaluating the balance or preference between the use of a home language (L1) and an additional language, English (L2), provides a quantitative dimension to the scale. Finally, the measurement scale should capture the depth of connection between social-cultural context and language identity. This involves assessing the extent to which an individual’s language identity is intertwined with various socio-cultural contexts. By incorporating these dimensions into the scale, we can create a comprehensive tool that not only measures language identity but also provides a rich understanding of the intricate interplay between language and socio-cultural environments. We will conduct focus group discussions (Stage 1A) to understand the background of participants’ language identity, then move on to in-depth interviews (Stage 1B) to capture detailed information that was not found from focus group discussions. 

### 3.1. Stage 1A: Focus Group Discussions

#### 3.1.1. Study Design

Hybrid semi-structured focus group discussions will be conducted both in-person and virtually using English at a university located in the San Francisco Bay Area. Although we will use English as a common shared language, participants will be encouraged to share and explain words or phrases as desired in any additional languages. The space will be created to make participants comfortable sharing their home languages, words, phrases, and ideas. The average duration of each focus group discussion will be approximately 90 to 120 min to provide sufficient time for participants to interact with each other. From the peer group dynamics, participants will be encouraged to share their thoughts to understand how they use language in different sociocultural contexts.

#### 3.1.2. Recruitment

**Figure 2:**
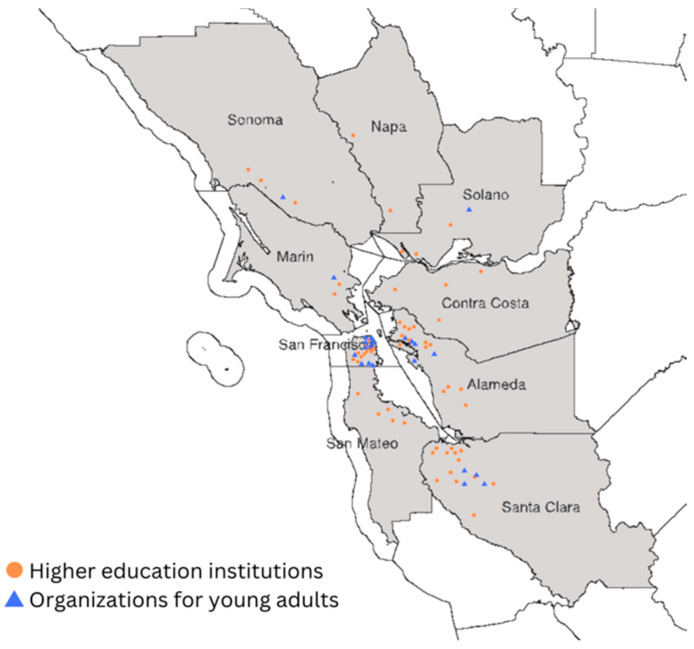
Distribution of target educational institutions and organizations.

We will use a purposive sampling strategy to identify and recruit target participants. The inclusion criteria of the participants for this study are: (1) Asian or Asian American young adults, (2) they were children or adolescents aged 5–17 years when coming to the U.S., (3) they came with their parents (1st generation), (4) they have lived in the U.S. for at least 12 months, (5) their current ages are 18–29 years, and (6) they currently live in Santa Clara County. The term 1.5 generation implies that the individual grew up in a country outside the U.S., and thus was exposed to one or more languages that differ from American English. This also suggests that they are able to understand and/or communicate (e.g., gestures) in a language/culture that differs from American English norms. We will send email invitations to university or college-wide offices and buildings (e.g., international student offices, undocumented student service centers, financial aid and scholarship offices, and dormitories) and various student clubs or organizations for young adults (e.g., academic and honorary organizations, cultural and religious organizations, special interest organizations, and non-profit organizations) located in Santa Clara County (Figure 2), and post the invitations on their social media and university mobile app. A purposive and snowball sampling strategy will be employed, recruiting participants who express their willingness to participate in a focus group discussion. Participants will be contacted via email and invited to advertise and disseminate qualitative study information to potential other participants through social media (e.g., Instagram, X, and LinkedIn). 

#### 3.1.3. Data Collection

A total of approximately 40 participants will be recruited to join focus group discussions. Each focus group discussion will seat up to 8 participants, for a total of 5 focus groups. As an incentive, a $40 gift card will be provided to all participants. The discussions will be conducted using a moderator’s guide addressing key initial questions, such as ‘How does speaking more than one language contribute to your sense of racial/ethnic identity?’, ‘Do you believe that English is “replacing” your home language(s) in any way? If so, how does this make you feel in relation to your sense of identity?’, and ‘Do the languages you use affect your sense of belonging as an immigrant? If so, why?’. Each of the focus group discussions will be audio-recorded and transcribed verbatim. To gain initial transcriptions, Otter.ai Business (Otter, Los Altos, CA, USA), a speech-to-text transcription software, will be used. Undergraduate research assistants will review those initial transcriptions to finalize verbatim transcriptions. Transcript preparation will also include redacting identifying information. The recordings will be viewed only by the research team and not shared.

#### 3.1.4. Data Analysis Process

Following transcription preparation, we will use qualitative analysis software, NVivo (QSR International, Pty, Ltd., Doncaster, Australia), to analyze five verbatim transcripts. Such software will facilitate the organization and coding of main codes and subcodes, with relevant quotes linked to each of those codes. Qualitative analysis will proceed in two methods: deductive and inductive approaches [69]. First, we will use deductive coding from the predefined topics outlined in the focus group guide, which will help ensure that key areas of predetermined interest are systematically addressed. Table 1 presents the anticipated deductive codes and their subcodes. Next, we will conduct an inductive coding process to capture newly discovered themes and insights that emerge from the data, which will be integrated wherever appropriate. Two members of the research team will independently identify codes from the first two transcripts, followed by a cross-checking process to reach a consensus on the preliminary coding frame. This collaborative approach will enhance the reliability of our coding. After completing this initial phase of the inductive coding process, we will examine response patterns within and across codes to identify overarching themes. As we analyze the remaining transcripts, we will iteratively refine the coding frame through ongoing inductive process, allowing for the integration of emerging insights. The final coding tree will consist of themes, categories, and codes, providing a comprehensive framework for understanding the qualitative data. Regarding the selection of key quotes that represent each of the main codes and subcodes for reporting, we will identify them across all five focus groups to ensure that opinions and thoughts from each group are equally represented. 

#### 3.1.5. Primary Outcome

We hypothesize that focus group discussions will provide preliminary information on how to separate language identity from other overlapping constructs, primarily language proficiency and acculturation in prior literature. The knowledge gained from these focus groups will be a useful synopsis for further understanding in the subsequent in-depth interviews.

### 3.2. Stage 1B: In-Depth Interviews

#### 3.2.1. Study Design

Semi-structured 1-on-1 in-depth interviews will be conducted using English in person or online at the university in the San Francisco Bay Area. The interviews will emphasize capturing detailed personal experiences that emerge from comprehensive one-on-one conversations. Some of the most common Asian languages in the Bay Area are Mandarin, Cantonese, Tagalog, Vietnamese, Korean, Hindi, Tamil, and Japanese [70,71]. Through a multilingual research team, we will allocate time and resources for translators and student interviewers with specific language skills to provide translation services as needed. The expected duration of each interview will be 60–90 min. An online interview will be conducted via Zoom (Video Communications, San Jose, CA, USA) for those who are not able to meet in person. To protect participants’ confidentiality, we will inform them before beginning the interview that they can choose to use their nickname or a pseudonym in Zoom as well as choose to turn off their video. 

#### 3.2.2. Recruitment

We will recruit a subset of focus group (Stage 1A) participants for the in-depth interviews in this Stage. It is important that focus group participants also join an in-depth interview to achieve consistent qualitative analysis with the same subjects. We will thoroughly explain to participants about the importance of their continued engagement and commitment to the study. Additionally, after focus group discussions are completed, we will email preliminary findings (e.g., sharing an executive summary) as well as regular updates (e.g., news articles about Asian/Asian American immigrant young adults) to participants for pursuing a high retention rate for in-depth interviews. We aim for approximately 50% of participants from focus group discussions to sequentially join in-depth interviews. If there are more than 50% of subjects who withdraw from the study after focus group discussions, we will contact other potential in-depth interview participants. Approximately a total of 20 young adults will be recruited; thus, there will be 20 in-depth interviews. As an incentive, a $40 gift card will be provided to all participants. 

#### 3.2.3. Data Collection and Data Analysis Process

The same approach as in Stage 1A will be used. The only difference is there will be 20 in-depth interviews. Table 2 outlines the main codes and their subcodes derived from deductive reasoning, and inductive reasoning will be incorporated from the findings from in-depth interviews. 

#### 3.2.4. Primary Outcome

We will gather detailed information about individual language identity through in-depth interviews, which will focus on the specific experience of interview participants. This information may either reinforce, support, or present discordance with the findings from focus group discussions. 

## 4. Stage 2: Quantitative Analysis

Using language identity measurement scale (Stage 2A), a cross-sectional online survey (Stage 2B) and multivariate multiple linear regression model (Stage 2C), we will determine the extent to which perceived language proficiency and language identity are associated with psychological well-being and mental health among 1.5 generation Asian/Asian American young adults.

### 4.1. Stage 2A: Language Identity Measurement Scale Development

#### 4.1.1. Study Design

In Stage 2A, our objective is to craft a novel language identity measurement scale by leveraging qualitative insights obtained from both focus group discussions and in-depth interviews. The process of developing this instrument involves a detailed analysis of the qualitative data gathered and identification of the most common themes, which will serve as the foundation for generating scale items. Following the methodology for scale development validated by DeVellis [72], we will meticulously transform and integrate these qualitative findings into a comprehensive and robust measurement scale. 

#### 4.1.2. Primary Outcome

This scale is intentionally designed to stand apart as a distinct construct from the conventional notion of perceived language proficiency. This strategic approach ensures that our measurement scale captures the nuanced facets of language identity, contributing to a more refined and accurate understanding of this complex construct.

### 4.2. Stage 2B: Cross-Sectional Online Survey

#### 4.2.1. Study Design

We will design a cross-sectional quantitative, online questionnaire written in English. We will use validated instruments where possible, and include the new measurement scale for language identity developed in Stage 1, to measure independent and dependent variables of interest, including perceived language proficiency, language identity, anti-Asian hate crime victimization, ethnocultural identity confusion, acculturative stress, racism and discrimination, psychological well-being, and mental health, as well as demographic information among 1.5 generation Asian/Asian American young adults. 

#### 4.2.2. Sample Size Justification

We will reach out to approximately 600 survey participants (1.5 generation Asian/Asian American immigrant young adults) as a sampling frame, which will allow us to estimate the outcome of interest from the representative of the target population. Using G*Power 3.1 [73], given an anticipated F-squared effect size of 0.15, α of 0.05, Power (1–β) of 0.8, and predictors (independent variables, including mediator variables), the minimum required sample size for finding statistical significance is 146 participants, which will give us a response rate of 24.3% (146/600). This sample size expectation may be adjusted once we complete the survey design and make final decisions on instrumentation. To increase the completion rate, participants will be recontacted if they initiated but did not complete the survey. 

#### 4.2.3. Recruitment

The same recruitment strategy will be used as in Stage 1, but the catchment area will be expanded to the San Francisco Bay Area. Because the criteria are very specific to variables associated with lower response rates (e.g., race, exposure to English, age, family type, the period of residence in the U.S., and geography), there could be challenges in reaching the desired sample size; thus, our online survey will be distributed to all 57 higher education institutions—across the University of California, California State University, the California Community Colleges, private nonprofits, and private for-profits—and 23 identified organizations for young adults located in 9 counties of the Bay Area (Alameda: 19, Contra Costa: 4, Marin: 3, Napa: 2, San Francisco: 22, San Mateo: 5, Santa Clara: 17, Solano: 4, and Sonoma: 4) (Supplementary Materials Table S1). A $10 gift card will be provided to participants who complete the online survey.

#### 4.2.4. Data Collection

We will use Qualtrics^xm^ (Qualtrics International Inc., Provo, UT, USA) to collect online survey data, and then store quantitative data in Google Shared Drive (Google Inc., Mountain View, CA, USA), which are both exclusively for research team members to protect data confidentiality. 

#### 4.2.5. Measurement

The survey will consist of 9 main categories: (1) demographic information (confounders), (2) self-perceptions of language proficiency in the languages that participants are exposed to (independent variable I), (3) language identity (newly developed from Stage 1; independent variable II), (4) ethnic identity (mediator I), (5) acculturative stress (mediator II), (6) racism and discrimination (mediator III), (7) anti-Asian hate crime victimization (moderator), (8) psychological well-being for satisfaction/optimism levels of adjustment in the U.S. (dependent variable I), and (9) mental health (dependent variable II). For demographic information, the country of origin, current age, age of arrival, length of time in the U.S., gender, socioeconomic status, family type, place of residence, religion, educational attainment, family background, and learning environment will be collected. Participants’ perceived language skills in their languages will be measured through a published self-report classification tool [74] and the Language Experience and Proficiency Questionnaire (LEAP-Q) [75]. To assess language identity, we will use our version of a language identity measurement scale developed in Stage 1. Ethnocultural identity confusion will be measured using the Ethnic Identity Scale (EIS) [76] and the Multigroup Ethnic Identity Measure-Revised (MEIM-R) [77]. To measure acculturative stress due to L2 learning for adjustment to U.S. society, the Demands of Immigration Scale (DIS) [78] will be used, which measures demands related to immigration, occupational adjustment, language accommodation, discrimination, and resettlement issues. For measuring racism and discrimination, the +20-item Perceived Discrimination Scales [79] will be used. Regarding anti-Asian hate crime victimization, we will use the National Crime Victimization Survey (NCVS) [80], modifying it specifically for Asians/Asian Americans. The Ryffs Scales of Psychological Wellbeing-54 [81] will be used for well-being measurement. Mental health disparities will be assessed using the Center for Epidemiologic Studies-Depression Scale (CES-D) [82], the Patient Health Questionnaire-9 (PHQ-9) [83], and the Generalized Anxiety Disorder-7 (GAD-7) [84]. To measure participants’ experiences related to the pandemic, we will add a quantitative measurement scale, II. Coronavirus (COVID-19) Pandemic section from the Baylor Religion Survey (Wave 6, 2021) [85], to our survey. Table 3 displays variable types, constructs, and measurement names. 

#### 4.2.6. Data Analysis

To analyze quantitative data collected from the online survey for a descriptive cross-sectional study, we will use a quantitative analysis software, Stata/MP 18.0. The data used for analysis will be separated from identifying information (i.e., email addresses). Only an ID number will be used on surveys. Our analysis will primarily aim to ascertain if language identity and perceived language proficiency are separate concepts and to measure quantitative variables related to interests and demographic information among 1.5 generation Asian/Asian American young adults. 

#### 4.2.7. Primary Outcome

We expect to design the survey with relevant measurements and collect data from the target sample size. We will collect data regarding both perceived language proficiency and language identity. In addition, designing the online survey will allow us to measure the association between perceived language proficiency/language identity and psychological well-being/mental health disparities of 1.5 generation Asian/Asian American immigrant young adults in the Bay Area, California for the next step, Stage 2B. 

### 4.3. Stage 2C: Multivariate Multiple Linear Regression Analysis

#### 4.3.1. Study Design

**Figure 3:**
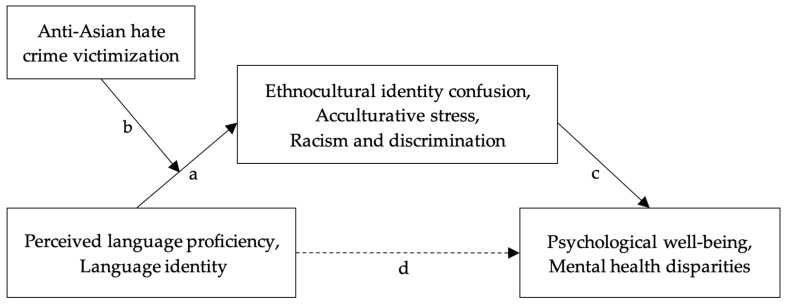
Possible path model of the association between perceived language proficiency and language identity and health disparities and mental health conditions with mediators.

Using the multivariate multiple linear regression model through the Ordinary Least Squares (OLS) regression technique, we will measure two dependent variables, psychological well-being and mental health, and their relationship to two independent variables, perceived language proficiency and language identity in multilingual individuals among our target population. To explore the hypothesis that language identity is associated with individual differences in psychological well-being and mental health disparities, we will examine the extent to which acculturative stress (cultural disorientation), and racism and discrimination mediate this association. Figure 3 demonstrates the possible finding that young adults who report weaker self-perceived language skills in the dominant language (English) and a lower sense of language identity (L1 and L2) (independent variables) will be more susceptible to ethnocultural identity confusion, acculturative stress, and racism and discrimination, leading to separation or marginalization from society (mediators) (Path a), which would ultimately result in disparities in psychological well-being and mental health (dependent variables) (Path c). Anti-Asian hate crime victimization will be treated as a moderator (effect-modifier) (Path b), acting between the independent variable and the mediator. Additionally, we will investigate a possible direct pathway between the independent and dependent variables (Path d). Using the product-of-coefficients approach, we will explore the indirect effects of an independent variable on a dependent variable through a mediator variable as a starting point. Then, we will use a sophisticated method, the bias-corrected bootstrapping, to obtain more accurate estimates of the significance of mediated effects in the proposed sample (n = 146).

#### 4.3.2. Data Analysis

The same approach as the cross-sectional online study for the quantitative analysis of OLS will be used. 

#### 4.3.3. Primary Outcome

We aim to disentangle the previously confounded constructs from the existing perceived language proficiency and the newly developed language identity scales from Stage 1 by employing those two distinct measures. We will assess whether the two constructs, perceived language proficiency and language identity, make distinct contributions to psychological well-being/mental health. This approach will allow us to examine the potential differential effects of these two language-related constructs on psychological well-being and mental health disparities in 1.5 generation Asian/Asian American immigrant young adults in the Bay Area, California. Analyses of the effects of potential mediator variables, including ethnocultural identity confusion, acculturative stress, racism, and discrimination, will be presented. Additionally, we will measure the moderating effect of the variable anti-Asian hate crime victimization, which may influence the magnitude of the relationship between the independent and moderator variables.

## 5. Stage 3: Qualitative Analysis

Subsequent in-depth interviews will be conducted to flesh out contextual information and patterns of experience about the cross-sectional information examined in the quantitative analysis, using a subsample of quantitative survey respondents in Stage 2. Based on their survey responses, we will seek to gain an in-depth understanding of experiences with acculturation and discrimination and how these experiences relate to their own perceptions of language skills and psychological well-being/mental health (Stage 3A). An analysis of qualitative data will provide further context around the statistical results of the quantitative analysis [86]. Translation support for those who note that they prefer a bicultural or bilingual interviewers will be provided, just as in Stage 1. 

### 5.1. Stage 3A: Follow-Up In-Depth Chronological Interviews

#### 5.1.1. Study Design

The follow-up semi-structured, chronological interviews (life history format) will be conducted sequentially with a subset of survey participants using their preferred languages. Interviews are expected to have the same duration as in Stage 1B, lasting between 60 and 90 min. These interviews aim to enhance our understanding of the quantitative association between perceived language proficiency/language identity and psychological well-being/mental health disparities. Through gathering unique information from individual narratives, these interviews will provide data on the actual individual trajectories illustrating how language proficiency and language identity are connected to mental health and psychological well-being. The methods for conducting interviews will be the same as Stage 1B. 

#### 5.1.2. Recruitment

The recruitment area will be expanded from Santa Clara County, used in Stage 1, to the San Francisco Bay Area, Northern California, as it is for the survey. There will be two different groups: (1) a group that reported mental health consequences, and (2) a group did not report mental health issues. Approximately 20 young adults who completed the online survey will be recruited, and there will be 20 in-depth interviews, 10 participants from one group and the other 10 participants from the other group. We will choose participants selectively to obtain well-balanced samples that reflect our various demographic variables, such as age range and the distribution of countries of origin [68]. An incentive of $40 gift cards will be provided to all participants, and additionally, to encourage participants to join an interview, a transportation voucher of up to $30 will be provided for those who live farther than 57 miles from the study location—this is the average distance from all 80 target institutions/organizations to the main study location.

#### 5.1.3. Data Analysis Process

We will use the same approach as in Stage 1 using a deductive/inductive approach. Table 4 shows the deductive approach in this planning stage, to which inductive approach coding will occur after the completion of in-depth interviews. These transcripts, however, will be longer and more extensive than in Stage 1 because we will pursue life history, asking participants to discuss a chronology of life (and language use) as a 1.5 generation immigrant. 

#### 5.1.4. Primary Outcome

We expect that in-depth interviews will confirm the association between perceived language proficiency/identity and psychological well-being/mental health disparities identified in Stage 2. In-depth interviews will strengthen the findings through unique individual narratives that were not captured in Stage 2. 

### 5.2. Ethical Considerations

We will use Qualtrics^xm^ (Qualtrics International Inc., Provo, UT, USA), Stata/MP 18.0 (StataCorp, College Station, TX, USA) and NVivo (QSR International, Pty, Ltd., Doncaster, Australia) to collect, manage, and analyze data, and then store them in the Google Shared Drive (Google Inc., Mountain View, CA, USA), which are both exclusively for research team members to protect data confidentiality. There will be four consent notices (written consent electronic English documents, participant’s signature not required). Once they confirm their intent to participate by replying to our request via email, a separate consent form PDF document for a focus group discussion will be emailed to them as a reminder, at least 24 h prior to each discussion (Stage 1A). The in-depth interview consent notice will be administered right after the focus group discussions (Stage 1B). There will be a standard consent notice for an online survey, embedded at the beginning of the Qualtrics survey as electronic texts (Stage 2A). Participants’ email addresses used for the recruitment of focus group and in-depth interview participants (a subsample of quantitative survey participants) will be collected as a sole identifier. Email information will be kept in a separate encrypted file. Those who fill in the online survey will be asked whether they can join an in-depth interview (Stage 3A). A standard consent form will be provided to participants at least 24 h prior to an in-depth interview.

## 6. Discussion

### 6.1. How the Proposed Research Would Shift Current Research Paradigms

This proposed research and its methodology is innovative because it will be the first to investigate how the constructs of perceived language proficiency and language identity can link to a sense of belonging, psychological well-being, and mental health among multilingual 1.5 generation Asian/Asian American immigrant young adults, who are vulnerable to becoming victims of anti-Asian hate crimes. Moreover, immigrant-tailored social adjustment strategies for feelings of acceptance in American society associated with learning an L2 have not been substantially discussed. This specific circumstance of multilingualism presents unique sociocultural benefits (i.e., a multilingual and multicultural worldview), but also unique challenges to learning more than one language under strong societal norms and pressures when there is a new dominant language and new culture. Ultimately, we expect this study will be the cornerstone of establishing culturally tailored multilingual education for Asian/Asian American immigrant young adults’ strong sense of acceptance, inclusion, identity, and psychological well-being/mental health in the U.S. 

### 6.2. Contribution to the Field of Mixed Methods Research

Collectively, this study protocol uses an innovative approach because it uses a newly developed three-step mixed methods approach (Stage 1: qualitative, Stage 2: quantitative, and Stage 3: qualitative), representing a significant advancement from traditional explanatory or exploratory sequential designs. By integrating qualitative and quantitative analyses in a systematic and iterative manner, this study addresses several methodological gaps and offers new insights that advance the field. Additionally, in reflecting upon the literature reviewed in the background section, this study bridges the gap between qualitative explorations of language identity and quantitative assessments, providing a coherent and integrated methodological approach. It demonstrates the value of sequential mixed methods designs in developing and validating measurement scales, and in elucidating complex relationships between psychosocial constructs.

Overall, this study advances the field of mixed methods research by offering a multiple-step methodological framework for studying language identity and its relationship with mental health among 1.5 generation Asian/Asian American immigrant young adults in one state in the U.S., with future hopes to further validate our sample to broader U.S. 1.5 generation Asian/Asian American population. The findings of this study prompt a reevaluation of how language identity is conceptualized and measured, ultimately contributing to a deeper and more comprehensive understanding of identity formation in multicultural and multilingual settings.

### 6.3. Limitations

This research encounters a few limitations. Firstly, our inclusion criteria for recruiting potential participants who fit the definition of the 1.5 generation are stringent, requiring additional effort to identify suitable candidates. Specifically, participants must be Asian or Asian American young adults residing in the San Francisco Bay Area, who arrived in the U.S. with their first-generation parents between the ages of 5 and 17, have resided in the U.S. for at least 12 months, and currently fall within the age range of 18 to 29 years. While the anticipated number of 40 participants in the focus group discussions in Stage 1A and the minimum sample size of 146 participants in Stage 2B align with the consideration of potential challenges in identifying eligible participants, they may still present certain difficulties in fully validating this long-term protocol. Additionally, achieving thematic saturation and managing potential subjective variability might be areas to carefully monitor with the anticipated sample size for the Stage 1A focus group discussions and the Stage 2C survey analyses. Secondly, the geographic scope of our participant pool is confined to the San Francisco Bay Area in California, thereby limiting the representativeness of the broader 1.5 generation Asian/Asian American young adult population in California and across the U.S. A more geographically diverse sample can be considered throughout the study protocol to increase the validity of the study. 

### 6.4. Recommendations for Future Mixed Methods Inquiry

By moving beyond traditional exploratory or explanatory sequential designs to a three-step sequential mixed methods design, researchers can maximize the strengths of both qualitative and quantitative methods, thereby advancing knowledge and understanding in their respective fields. Researchers and methodologists are encouraged to look into this approach to other research contexts, recognizing the value of integrating qualitative and quantitative analyses in a sequential and iterative manner.

## Figures and Tables

**Table 1:** Stage 1A: Deductive approach for focus group discussions.

Main Code	Subcode
Background	preliminary context of using one language compared to the other language, relative connection between language usage and language identity
Language proficiency	perceived language proficiency and its overall relationship with participants’ background in the group
Language identity	language identity separated from language proficiency
Acculturation	sense of belonging and cultural experiences/preferences relevant to language proficiency and language identity

**Table 2:** Stage 1B: Deductive approach for in-depth interviews.

Main Code	Subcode
Language proficiency	individual English learning and environment from the home country, early-life English education, individual perceived language proficiency, individual experience in the context of language usage, continued discussion from Stage 1A
Language identity	individual experience and context for establishing language identity, individual ethnocultural identity, continued discussion from Stage 1A
Acculturation	individual sense of belonging, individual cultural experiences/preferences relevant to language proficiency and language identity, continued discussion from Stage 1A
Findings from Stage 1A	the degree of concordance, discordance, or expansion with Stage 1A using more detailed subcodes
Detailed codes	suggestions and feedback from participants for measurement scale development

**Table 3:** Stage 2B: The use of constructs and their measurements.

Variable Type	Construct	Measurement Name
Demographic information (confounders)	Not applicable	Not applicable
Independent variable I	Perceived language proficiency	Self-report classification tool [74] and Language Experience and Proficiency Questionnaire (LEAP-Q) [75]
Independent variable II	Language identity	Newly developed measurement scale from Stage 1
Mediator I	Ethnic identity	Ethnic Identity Scale (EIS) [76] and Multigroup Ethnic Identity Measure-Revised (MEIM-R) [77]
Mediator II	Acculturative stress	Demands of Immigration Scale (DIS) [78]
Mediator III	Racism and discrimination	+20-item Perceived Discrimination Scales [79]
Moderator	Anti-Asian hate crime victimization	National Crime Victimization Survey (NCVS) [80]
Dependent variable I	Psychological well-being	Ryffs Scales of Psychological Wellbeing-54 [81]
Dependent variable II	Mental health	Center for Epidemiologic Studies-Depression Scale (CES-D) [82], Patient Health Questionnaire-9 (PHQ-9) [83], and Generalized Anxiety Disorder-7 (GAD-7) [84].

**Table 4:** Stage 3A: Deductive approach for in-depth life history interviews.

Main Code	Subcode
Multilingualism	perceived language proficiency, category for use (when they use what)
Identity	perceived language identity related to actual language usage, ethnocultural identity
Anti-Asian hate crime victimization	experience before, during, and after COVID-19
Acculturation	fear, guilt, mood, acculturation stress, cultureshock, homesickness, emotional neglect
Racism/Discrimination	experience with anti-Asian hate crimes, perceived hatred/rejection/discrimination
Psychological well-being	relationship with others, happiness, personal growth, self-acceptance, purpose in life, environment, social support, accomplishment, life satisfaction
Mental health disparities	self-deprecation, stress, anxiety, insomnia, addiction, substance abuse, depression, post-migration trauma, relation to language identity
Opportunities	benefits of multilingual, confidence in life, resource sharing
Heading for the future	asking for needed support, intervention programs for the future studies

## Data Availability

Data sharing is not applicable to this article as no datasets were generated or analyzed during the current study.

## Supplementary Materials

The following supporting information can be downloaded at: https://www.mdpi.com/article/10.3390/ijerph21101311/s1, Table S1. List of higher education institutions in the San Francisco Bay Area.
