# Supplementary material for: Mixed Methods Study Protocol: Language Identity, Discrimination, and Mental Health among Multilingual 1.5 Generation Asian/Asian American Immigrant Young Adults
Source: Int J Environ Res Public Health. 2024 Sep 30;21(10):1311. doi: 10.3390/ijerph21101311 (PMC11507530; doi:10.3390/ijerph21101311)
Supplement: Supplementary file 1 [file ijerph-21-01311-s001.zip › ijerph-3169242-supplementary.pdf]

International Journal of
Environmental Research and Public Health

MDPI

Supplementary Materials

Mixed Methods Study Protocol: Language Identity, Discrimination, and Mental Health among Multilingual 1.5 Generation Asian/Asian American Immigrant Young Adults

Chulwoo Park [1,*], Mark Edberg [2], Janet Y. Bang [3] and Avizia Y. Long [4]

[1] Department of Public Health and Recreation, San José State University, San Jose, CA 95192, USA
[2] Department of Prevention and Community Health, Milken Institute School of Public Health, George Washington University, Washington, DC 20052, USA; medberg@gwu.edu
[3] Department of Child and Adolescent Development, San José State University, San Jose, CA 95192, USA; janet.bang@sjsu.edu
[4] Department of World Languages and Literatures, San José State University, San Jose, CA 95192, USA; avizia.long@sjsu.edu
* Correspondence: charles.park@sjsu.edu

Table S1. List of higher education institutions in the San Francisco Bay Area.

Phase	Counties	Higher Education Institutes (57)	Organizations for Young Adults (23)
1, 2, 3	Santa Clara (17)	▪ CSU (1): San Jose State University<br>▪ CCC (7): De Anza College, Evergreen Valley College, Foothill College, Gavilan College, Mission College, San Jose City College, West Valley College<br>▪ PN (4): International Technological University, Palo Alto University, Santa Clara University, Stanford University<br>▪ PFP (1): University of Silicon Valley	Asian Americans for Community (AACI), National Asian Pacific Islander Desi American Panhellenic Association (SJSU), Vietnamese American Roundtable, Vietnamese American Service Center (VASC)
2, 3	Alameda (19)	▪ UC (1): University of California Berkeley<br>▪ CSU (1): California State University East Bay<br>▪ CCC (7): Berkeley City College, Chabot College, College of Alameda, Laney College, Las Positas College, Merritt College, Ohlone College<br>▪ PN (4): Holy Names University, Lincoln University, Mills College at Northeastern University, Samuel Merritt University<br>▪ PFP (1): SAE Expression College	Asian Pacific Islander Community Collaborative, Community Health for Asian Americans, East Bay Asian Youth Center, Korean Community Center of the East Bay, Vietnamese American Community Center of the East Bay
2, 3	Contra Costa (4)	▪ CCC (3): Contra Costa College, Diablo Valley College, Los Medanos College<br>▪ PN (1): Saint Mary's College of California	
2, 3	Marin (3)	▪ CCC (1): College of Marin<br>▪ PFP (1): Dominican University of California	Marin Asian Advocacy Project
2, 3	Napa (2)	▪ CCC (1): Napa Valley College<br>▪ PN (1): Pacific Union College	
2, 3	San Francisco (22)	▪ UC (2): University of California Hastings College of the Law, University of California San Francisco<br>▪ CSU (1): San Francisco State University<br>▪ CCC (1): City College of San Francisco<br>▪ PN (6): California College of the Arts, California Institute of Integral Studies, Golden Gate University, Hult International Business School, San Francisco Conservatory of Music,	Asian Pacific American Community Center, Asian Women's Shelter, Chinatown Community Development Center, Chinese Culture Center of SF, Donaldina Cameron House, Filipino Community Center, Japanese Community Youth Council, Japanese Cultural and Community Center of Northern California, Korean American Community Foundation, Korean Center, Inc., Pacific Asian American Women Bay Area Coalition

		University of San Francisco<br>■ PFP (1): Academy of Art University	
2, 3	San Mateo (5)	■ CCC (2): Cañada College, College of San Mateo<br>■ PN (3): Menlo College, Notre Dame de Namur University, Skyline College	
2, 3	Solano (4)	■ CSU (1): California State University Maritime Academy<br>■ CCC (1): Solano Community College<br>■ PN (1): Touro University California	Chinese American Association of Solano County
2, 3	Sonoma (4)	■ CSU (1): Sonoma State University<br>■ CCC (1): Santa Rosa Junior College<br>■ PFP (1): Empire College	Asian American and Pacific Islander NAMI
